# Nonsteroidal anti-inflammatory drugs (NSAID) do not increase blood loss or the incidence of postoperative epidural hematomas when using minimally invasive fusion techniques in the degenerative lumbar spine

**DOI:** 10.3389/fsurg.2022.1000238

**Published:** 2022-11-04

**Authors:** Wolfgang Senker, Stefan Aspalter, Wolfgang Trutschnig, Jörg Franke, Andreas Gruber, Harald Stefanits

**Affiliations:** ^1^ Department of Neurosurgery, Kepler University Hospital, Johannes Kepler University, Linz, Austria; ^2^ Department of Mathematics, University of Salzburg, Salzburg, Austria; ^3^ Department of Orthopedic Surgery, Klinikum Magdeburg, Magdeburg, Germany

**Keywords:** minimally invasive spine surgery, lumbar fusion, blood loss, hematoma, non-steroidal anti-inflammatory drugs, NSAID

## Abstract

**Objective:**

Nonsteroidal anti-inflammatory drugs (NSAID) are essential in surgeons' armamentarium for pain relief and antiphlogistic effects. However, spine surgeons are concerned about the drugs' impact on coagulation, fearing hemodynamic instability due to blood loss and neurological complications due to postoperative hematoma. Furthermore, there are no clear guidelines for the use of these drugs.

**Materials and methods:**

In this retrospective subgroup analysis of a prospective observational study, we investigated 181 patients who underwent minimally invasive spinal fusions in degenerative lumbar spine pathologies. 83 patients were given NSAID perioperatively, 54 of which were female and 29 male. Of these patients who took NSAID, 39 were on NSAID until at least one day before surgery or perioperatively, whilst the others discontinued their NSAID medication at least three days before surgery. Differences in perioperative blood loss, as well as complication rates between patients with and without NSAID treatment, were investigated.

**Results:**

A significantly higher amount of blood loss during surgery and the monitoring period was encountered in patients whose spine was fused in more than one level, regardless of whether NSAID medication was taken or not and up until what point. Furthermore, it was found that taking NSAID medication had no effect on the incidence of postoperative epidural hematomas.

**Conclusion:**

Perioperatively taking NSAID medication does not increase blood loss or the incidence of postoperative hematoma in patients undergoing minimally invasive lumbar spinal fusion surgery.

## Introduction

Minimally invasive surgery (MIS) is thought to create a smaller corridor to the spine, resulting in less tissue injury. Furthermore, MIS is associated with reduced blood loss, faster recovery, and lower perioperative morbidity rates whilst yielding similar results to open procedures (1–5). Our study discusses the controversial subject of a possible elevated risk of bleeding associated with perioperative nonsteroidal anti-inflammatory drugs (NSAID) which are prescribed for their analgesic and antiphlogistic effects. The aim of this retrospective subgroup analysis of a prospective observational study, which is based on data from 187 patients, is to examine whether patients who undergo minimally invasive surgery (MIS) while taking NSAID are at risk of increased blood loss and incidence of postoperative hematoma compared to patients who do not receive NSAID treatment.

## Materials and methods

We obtained approval from the ethics committee of the Federal State of Lower Austria and registered the study at ClinicalTrials.gov (NCT01259960). Written consent of all patients was obtained to carry out the study. Of the 187 patients included in this research, 115 were female and 72 male. All patients were treated with one, two, three, or four level minimal invasive fusion. In 146 patients, additional decompression of the spinal canal was performed. Blood loss was defined as the primary endpoint. We recorded the amount of blood loss during surgery as well as during the monitoring period in the recovery unit and the postoperative period, the latter until the removal of the drainage. Volumes were measured and recorded in milliliters. As a secondary endpoint, we defined postoperative epidural hematomas. In the case of clinical suspicion of the presence of epidural hematomas, an MRI was performed. If the radiological findings described a postoperative epidural hematoma, we accordingly recorded this. We enrolled only patients in this study who regularly took NSAID as analgesics or antiphlogistics up until one day before surgery or perioperatively. Not all 187 patients were included in the analysis of this study. No information on NSAID intake was available for four patients, and for two patients, the information on blood loss (perioperative and monitoring) or drainage volume was missing. Thus, 181 relevant patients (111 female and 70 male) remained in this study.

### Surgical technique

After identifying the correct facet joint under fluoroscopy control, an incision was made 1.5 cm off the midline. Using a tubular retractor system, muscle tissue was sequentially dilated. After visualization of the facet joint and yellow ligament, percutaneous fusion was performed. In cases of spinal stenosis, a laminotomy and decompression were performed. For interbody fusion, a TLIF procedure (transforaminal interbody fusion) was followed. In nine patients, we did not implant an interbody device at every level because of the narrow disc space and the associated risk of fracturing the corresponding endplates. In four two-level fusion cases, we fused only one level with a TLIF cage. In four three-level fusion cases, we implanted two TLIF cages, and in one four-level fusion case, we inserted three TLIF cages.

### Statistical analysis

Statistical analyses were performed using the R package npmv. The nonpartest was used to test the null hypothesis that the underlying distributions in the groups under investigation coincided. Whenever portions were considered, the standard *k*-sample test for equality of proportions was used. Linear dependence of variables was determined by Pearson's correlation, while the correlation was quantified by Spearman's rank correlation. Statistical significance was assumed at a *p*-value of <0.05.

## Results

### Patient population

**Figure 1:**
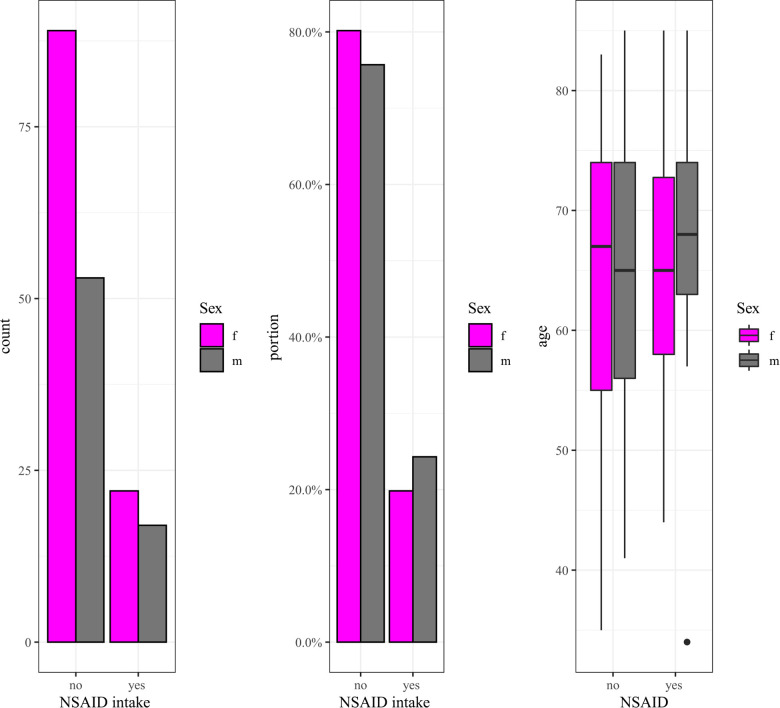
Number and percentage of patients in the NSAID and the non-NSAID group; age distributions in the two groups.

**Figure 2:**
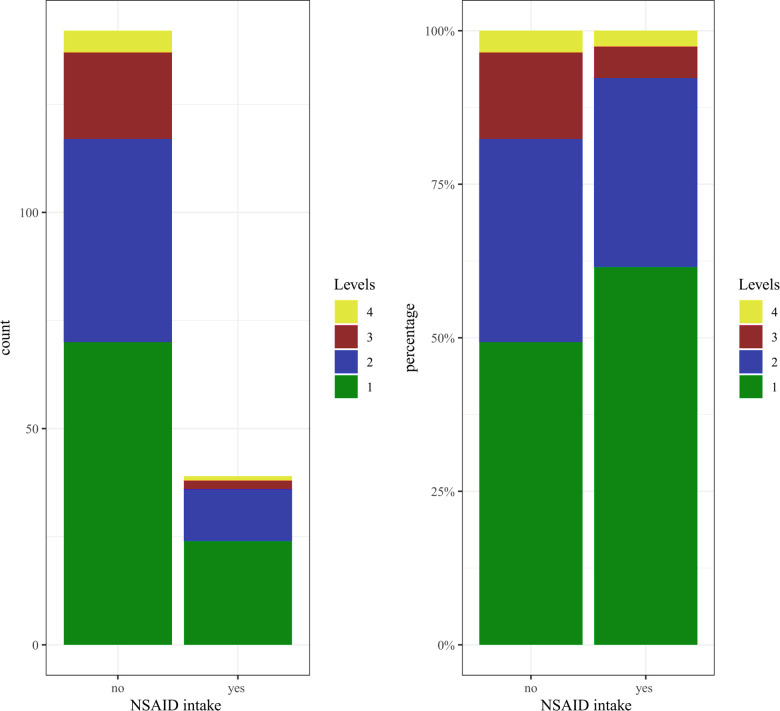
Distribution of the number of levels in the NSAID and the non-NSAID group (patients in the NSAID group tend to have fewer segments operated on than patients in the non-NSAID group).

Of the 181 patients under investigation, 83 (45.86%) received NSAID, 54 of which were female (48.65%) and 29 male (41.43%). Within this subset of 83 patients who had taken NSAID, 39 patients had taken the medication until at least the day before surgery or perioperatively. The remaining 44 patients had stopped taking NSAID as recommended three days before surgery. In the following section, the expressions “NSAID intake” or “patients in the NSAID group” refer to those patients who had taken NSAID until at least the day before surgery or perioperatively. All other patients (*n* = 142), including 44 individuals who had discontinued NSAID at least three days before surgery, are referred to as the “non-NSAID” group. Because age and blood loss/drainage volume are weakly positively correlated, we compared the age distribution of patients in the NSAID and the non-NSAID groups and found that they did not differ significantly (*p* = 0.58) (Figure 1). We further divided the patients into subsets of those with one fused level (“one-level” group) and those with two, three, or four fused levels (“two-plus” group). Patients in the NSAID group tend to have fewer levels operated on than patients in the non-NSAID group (*p* = 0.12) (Figure 2). We could not observe a difference in the proportion of NSAID-taking patients who had one, two, three, or four levels fused (*p* = 0.38).

### The impact of the number of fused levels on perioperative blood loss and drainage volumes

Information on the number of operated levels, blood loss (perioperative and monitoring), and drainage volumes were available for 183 of the 187 patients. In order to be able to analyze the impact of NSAID on these measurements, we had to exclude other confounding factors at first. To do so, we split the data into two groups of approximately similar size: 94 patients (51.37%) were fused in only one level (“one-level” group), and 89 patients (48.64%) were fused in two to four levels (“two-plus” group). Patients in the “one-level” group experienced a significantly lower amount of blood loss than in the “two-plus” group (*p* = 0.019; Table 1). Interestingly, this difference was only detectable in male patients (*p* = 0.06), whereas no significant differences were seen in the female cohort (*p* = 0.36). Furthermore, on average, the patients in the “one-level” group experienced significantly lower levels of blood loss by drainage than the patients in the “two-plus” group (*p* < 0.001; Table 2). Surprisingly, no differences between the two groups were found in male patients (*p* = 0.19). However, a statistically significant difference was detectable in female patients (*p* < 0.001). This led to the assumption that the number of fused levels has a high impact on both blood loss and drainage volumes but that this effect differs by gender. Consequently, we not only analyzed the impact of NSAID on blood loss separately for the subgroup of patients in the “one-level” group and those in the “two-plus” group but also looked at possible gender differences.

**Table 1:** Blood loss in patients in the “one-level” and “two-plus” level groups.

Sex	Levels	n	Mean blood loss (ml)
M	1	33	28,79
F	1	61	82,13
M	2+	38	143,29
F	2+	51	113,86

**Table 2:** Blood loss in patients in the “one-level” and “two-plus” level groups.

Sex	Levels	n	Mean drainage volume (ml)
M	1	33	120,91
F	1	61	108,52
M	2+	38	146,37
F	2+	51	196,47

### The impact of continued NSAID intake on blood loss (perioperative and monitoring)

In 181 patients (111 female and 70 male), the following variables were known: (I) number of fused levels, (II) blood loss (perioperative and monitoring), (III) drainage, and (IV) NSAID intake (Table 3). As mentioned previously, 39 of the 181 patients in this study were given NSAID medication until at least one day before surgery or perioperatively. However, there was no difference in perioperative blood loss or blood loss during the monitoring phase between patients who took NSAID and those in the “non-NSAID” group, neither in the “one level” group (*p* = 0.69) nor in the “two-plus” group (*p* = 0.74). Even when accounting for the impact of gender, we couldn't find any statistically significant differences concerning blood loss and NSAID intake between males and females in the “one level” and “two level” subgroups, although there was a slightly higher level of blood loss in women taking NSAID who were operated on two levels or more (Table 4; *p* = 0.06).

**Table 3:** Blood loss volume distributions and the possible impact of NSAID on mean and range of blood loss volume per subgroup.

NSAID	Levels	Sex	Mean blood loss (ml)	Minimum blood loss (ml)	Maximum blood loss (ml)	*n*
No	1	M	28,26	0	500	23
Yes	1	M	30	0	300	10
No	1	F	68,3	0	600	47
Yes	1	F	128,57	0	1000	14
No	2+	M	157,33	0	800	30
Yes	2+	M	42,86	0	300	7
No	2+	F	101,9	0	1050	42
Yes	2+	F	190,88	0	420	8

**Table 4:** Resulting *p*-values when testing for equal blood loss distributions for NSAID and non-NSAID patients in the four different subgroups formed by sex and (aggregated) number of segments.

Sex	Levels	*p*
M	1	0,84
F	1	0,86
M	2+	0,16
F	2+	0,06

### The impact of NSAID intake on blood loss *via* drainage

Drainage volumes did not differ between NSAID and “non-NSAID” patients, neither in the “one level” group (*p* = 0.59), nor in the “two plus” group (*p* = 0.12). Furthermore, when taking into account differences in gender and the number of fused levels, no statistically significant differences were observed (Table 5). However, slightly higher drainage volumes were found among female patients who took NSAID and underwent fusion of two or more levels (*p* = 0.06).

**Table 5:** Resulting *p*-values when testing for equal drainage distributions in patients with and without NSAID in the four different subgroups formed by sex and (aggregated) number of segments.

Sex	Levels	*p*
M	1	0,84
F	1	0,45
M	2+	0,79
F	2+	0,06

### The impact of NSAID intake on the incidence of epidural hematoma

Three of 181 patients encountered an intraspinal epidural hematoma (two females, one male). One further female patient, who suffered neurological disturbances postoperatively, was diagnosed with an extraforaminal hematoma. Thus, the incidence of an epidural hematoma was 2.2% in our series. All four patients with an epidural hematoma had to undergo revision surgery. However, only a single male patient was part of the NSAID group, whilst the females had discontinued NSAID medication ten days preoperatively, hadn't taken NSAID at all, or had not taken NSAID on a regular basis, respectively. The small subgroup size of patients with epidural hematomas doesn't allow for statistical analysis to be carried out. However, we assume that NSAID medication does not have a significant impact on the occurrence of epidural hematoma.

## Discussion

Prostaglandins are produced out of arachidonic acid, catalyzed by cyclooxygenase. NSAID works by blocking the synthesis of prostaglandins, thus mediating their analgesic, antipyretic and anti-inflammatory effects. Side effects of NSAID on the kidneys and stomach, or inhibition of thrombocyte aggregation, can be further consequences of this cascade (6). The detection of two different types of cyclooxygenase – COX 1 and COX 2 – helped to explain modes of action, which had, until then, seemed illogical (7). COX 1 produces prostaglandins which are responsible for the entire peripheral resistance, renal blood flow, and the renal elimination of sodium. COX 1 also catalyzes the production of protective prostaglandins in the stomach and the intestine. Moreover, it synthesizes thromboxane A2, which is responsible for the aggregation of thrombocytes and which makes it an interesting target for surgeons: blocking COX 1 leads to the suppression of thrombocyte aggregation, which in turn can result in greater bleeding. In contrast, COX 2 is primarily responsible for the production of prostaglandins during inflammatory reactions, which mostly occur during the course of pathophysiological processes mediated by Interleukin 1, Tumor necrosis factor-α, growth factor transformation, and others. To counteract only these effects whilst also reducing side effects, COX 2 selective inhibitors were introduced. Almost all NSAID which are used as painkillers or for antiphlogistic reasons block COX 1 or COX 2 in several dimensions (8, 9). Antiplatelet drugs such as acetylsalicylic acid (ASS) are widely used in primary and secondary prevention in atherosclerosis patients. This, in turn, caught the interest of surgeons due to possible bleeding complications. Korinth et al. presented the results of a survey of neurosurgeons on the topic of the discontinuation strategy of ASS (10). A broad range of days of discontinuation, seven days before surgery on average, was seen during the study. Two-thirds of the respondents felt that aspirin increased the risk of patients experiencing hemorrhagic complications, and more than half of the interviewed neurosurgeons reported having personally witnessed such problems during spinal operations. In a literature review, Gerstein et al. noted that the risk of perioperative bleeding associated with the continuation of aspirin medication is minimal in many operative procedures compared with the coincident thromboembolic risks associated with aspirin withdrawal. However, aspirin administration should be stopped in patients who are undergoing intracranial, middle ear, posterior eye, intramedullary spine, and possibly transurethral prostatectomy surgery (11). Soleman et al. investigated patients who underwent non-instrumented extradural lumbar spinal surgery (i.e., microscopic fenestration, recessotomy, foraminotomy, and sequestrectomy) under low-dose acetylsalicylic acid and without antiplatelet agents (12). They saw no statistical difference between the acetylsalicylic acid group and the control group and recommended the perioperative continuation of acetylsalicylic acid therapy, especially for the secondary prevention of perioperative complications in atherosclerotic patients. On the other hand, Park et al. (13) investigated the bleeding risk in patients undergoing one- or two-level lumbar spinal fusion surgery. They compared patients who discontinued aspirin medication more than seven days preoperatively, or between three and seven days preoperatively, with a group of patients who had not taken aspirin before surgery (control group). They found that if aspirin was discontinued more than seven days before surgery, there was no statistically significant difference in bleeding complications and blood loss compared with the control group. Cessation of aspirin medication three to seven days before surgery resulted in a significantly higher amount of drained blood and a longer duration of the indwelling of the drainage catheter than in the control group.

The use of NSAID as a painkiller or preoperative antiphlogistic therapy remains controversial. NSAID used as antiphlogistic or analgesic therapy are mostly COX 2 inhibitors. Nevertheless, they also show a limited amount of COX 1 inhibition. Consequently, the extent of the risk of bleeding associated with this medication remains subject to discussion. There is little literature examining this issue in spinal surgery. Park et al. looked at the possible increased blood loss in 106 patients who underwent at least two or more levels of lumbar fusion and who took NSAID (14). They found an increased level of blood loss in patients who took NSAID continuously before surgery compared with the non-NSAID group. In our patient group, blood loss during surgery, postoperative monitoring, and *via* drainage differed significantly between patients who had had one level fusion surgery and those who had had two and more level fusions. However, this effect differed by gender. In the literature, no sex difference has been described for lumbar spine surgery considering blood loss or complications like epidural hematomas (15, 16). While multi-level surgery is a known and logical appearing factor in an increase in blood loss (17), knowledge of the influence of sex seems to be lesser investigated. To our knowledge, no study focused specifically on this topic. In the cervical spine, a recent study by Wen et al. found a sex difference in blood loss in anterior cervical spine fusion (18), while a similar study by the same authors did not find any sex difference in blood loss in posterior lumbar fusion surgery (19). Due to our retrospective study design, no further conclusions or assumptions can be made on the reason for our findings. Further prospective studies are necessary to determine if there is a significant and clinically relevant sex difference in blood loss in posterior lumbar spine surgery.

We did not see a statistically significant difference between NSAID users (up until the day of or the day before surgery) and non-NSAID patients in any of the investigated subgroups.

We encountered three epidural hematomas which had to be revised, and one extraforaminal hematoma. Again, we found no statistically significant impact of NSAID medication on hematoma occurrence. We had previously investigated 33 patients aged 65 years or older who underwent minimally invasive spinal fusion surgery in another study (20). Interestingly, in this investigation, patients who preoperatively used NSAID as painkillers experienced greater levels of blood loss. We believe this contradiction to be the consequence of the low number of cases in the earlier study. The considerably higher number of cases investigated here gives this new study more weight.

Considering the usage of NSAID in spinal fusion, it also must be mentioned that NSAIDs are discussed as a factor responsible for the impairment of the fusion process. A review from 2017 found that the effect of NSAID to reduce fusion rates might only be present when using NSAID for a course of 2 weeks postoperative and more (21). Using NSAID in a short-term period postoperatively, this disadvantageous effect seems to be improbable. Also, other studies found a dose-dependent effect of NSAIDs on reduced fusion rates (22, 23). While we did not investigate these effects in our study, spine surgeons should consider not only possible effects on blood loss but also the fusion rates, especially when using NSAID for a long-term period postoperative.

Our study was limited due to the retrospective study design, and prospective, randomized controlled trials with a focus on NSAID and sex differences in blood loss after lumbar fusion surgery should be performed.

## Conclusions

MISS techniques minimize soft tissue damage, reduce blood loss and show less postoperative pain and result in a shorter hospital stay (1, 2, 4, 5, 24). We consider NSAID medication to have no counter-productive effects in minimally invasive fusion procedures up to four-level fusion with regard to blood loss levels or postoperative hematoma occurrence. Nevertheless, we recommend further prospective studies to confirm our results.

## Data Availability

The raw data supporting the conclusions of this article will be made available by the authors, without undue reservation.
